# Ra‐224 labeling of calcium carbonate microparticles for internal α‐therapy: Preparation, stability, and biodistribution in mice

**DOI:** 10.1002/jlcr.3610

**Published:** 2018-03-12

**Authors:** Sara Westrøm, Marion Malenge, Ida Sofie Jorstad, Elisa Napoli, Øyvind S. Bruland, Tina B. Bønsdorff, Roy H. Larsen

**Affiliations:** ^1^ Oncoinvent AS Oslo Norway; ^2^ Department of Tumor Biology, Institute for Cancer Research, The Norwegian Radium Hospital, Oslo University Hospital Oslo Norway; ^3^ Institute of Clinical Medicine University of Oslo Oslo Norway; ^4^ Department of Radiation Biology, Institute for Cancer Research, The Norwegian Radium Hospital Oslo University Hospital Oslo Norway; ^5^ Department of Oncology, The Norwegian Radium Hospital Oslo University Hospital Oslo Norway

**Keywords:** alpha therapy, calcium carbonate, intraperitoneal, microparticles, peritoneal carcinomatosis, radium‐224

## Abstract

Internal therapy with α‐emitters should be well suited for micrometastatic disease. Radium‐224 emits multiple α‐particles through its decay and has a convenient 3.6 days of half‐life. Despite its attractive properties, the use of ^224^Ra has been limited to bone‐seeking applications because it cannot be stably bound to a targeting molecule. Alternative delivery systems for ^224^Ra are therefore of considerable interest. In this study, calcium carbonate microparticles are proposed as carriers for ^224^Ra, designed for local therapy of disseminated cancers in cavitary regions, such as peritoneal carcinomatosis. Calcium carbonate microparticles were radiolabeled by precipitation of ^224^Ra on the particle surface, resulting in high labeling efficiencies for both ^224^Ra and daughter ^212^Pb and retention of more than 95% of these nuclides for up to 1 week in vitro. The biodistribution after intraperitoneal administration of the ^224^Ra‐labeled CaCO_3_ microparticles in immunodeficient mice revealed that the radioactivity mainly remained in the peritoneal cavity. In addition, the systemic distribution of ^224^Ra was found to be strongly dependent on the amount of administered microparticles, with a reduced skeletal uptake of ^224^Ra with increasing dose. The results altogether suggest that the ^224^Ra‐labeled CaCO_3_ microparticles have promising properties for use as a localized internal α‐therapy of cavitary cancers.

## 1 INTRODUCTION

The use of internal α‐emitters to treat cancer has attracted significant attention during the last decade. Their superior cytotoxicity and limited range in tissue corresponding to only a few cell diameters are properties that make them suitable for treatment of micrometastatic disease.^1^, ^2^ So far, the research efforts have culminated in 1 approved α‐emitting radiopharmaceutical, ^223^Ra‐dichloride (Xofigo^®^, Bayer), which is used for treatment of patients with skeletal metastases from castration‐resistant prostate cancer.^3^ Radium is an alkaline earth metal and thus chemically similar to calcium. This resemblance causes radium to target the bones, and to a larger degree, osteoblastic bone metastases.^4^ It is this property together with efficient cell kill from α‐particles that has made intravenous injection of ^223^Ra‐dichloride an effective therapy.^5^ The bone‐seeking property of radium was also exploited medically with ^224^Ra over many years (1950‐1990 and 2000‐2005),^6^, ^7^, ^8^, ^9^ although not in cancer but as palliative treatment of ankylosing spondylitis, a chronic inflammatory rheumatic disease.

**Figure 1:**
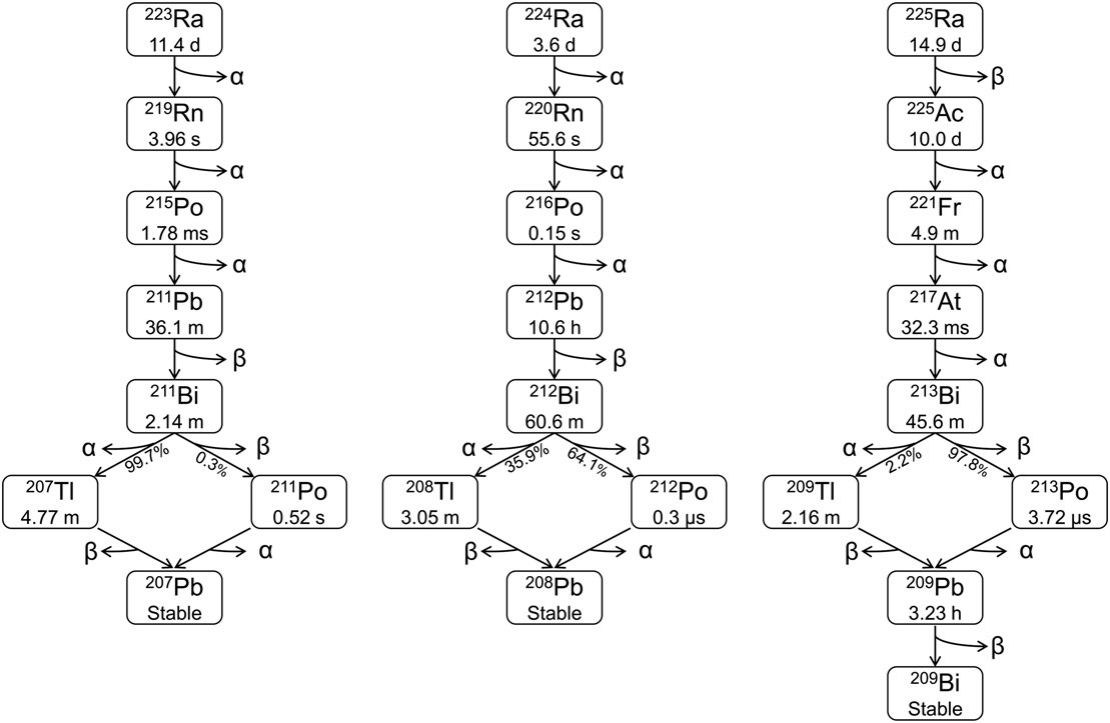
The decay chains of ^223^Ra, ^224^Ra, and ^225^Ra including details on each nuclides' half‐life and main mode of decay

Among the radium isotopes, there are 3 that stand out to be considered for biomedical applications as internal α‐emitters: ^223^Ra (*t*_½_ = 11.4 days), ^224^Ra (*t*_½_ = 3.6 days), and ^225^Ra (*t*_½_ = 14.9 days). Ra‐223 and 224 are by themselves α‐emitters, whereas ^225^Ra is a β‐emitter. A shared feature with the 3 series is that they decay via multiple α‐ and β‐emitting progeny with shorter half‐lives than their respective radium parent and an average of 4 emitted α‐particles per complete decay (Figure 1). The decay of each series releases a high total energy of 28 to 29 MeV, where more than 90% of the energy is associated with α‐emissions. This property enables delivery of therapeutically relevant doses at low administered activity levels. In addition, the 3 mentioned radium isotopes all have relatively long half‐lives which allows centralized production and quality control before shipment to the end user, such that challenges relating to production for radionuclides with short half‐lives can be avoided.

Despite their attractive properties for radionuclide therapy, the use of radium isotopes has so far mainly been limited to bone‐seeking applications. One reason for this is the lack of an appropriate bifunctional chelating agent for coupling of radium to targeting molecules. There have been a few studies of possible chelating agents,^10^, ^11^ but a bifunctional ligand that stably binds radium with control of daughter nuclides in vivo has yet to be presented. However, ^224^Ra has been used as a generator nuclide for its daughter ^212^Pb.^12^, ^13^ To fully exploit the therapeutic potential of radium isotopes for other purposes than bone targeting, alternative delivery systems are needed. One proposed strategy is to use nanoparticles or microparticles as carriers, and radium has been incorporated into the core of lanthanum phosphate nanoparticles,^14^ loaded into liposomes,^15^, ^16^ absorbed into the pores of nanozeolites,^17^, ^18^ and both intrinsically incorporated in the crystal structure and adsorbed onto the surfaces of hydroxyapatite nanoparticles and microparticles.^19^, ^20^, ^21^ Among these, all particles apart from liposomes are composed of inorganic materials. Another inorganic material that is promising for different biomedical applications is calcium carbonate (CaCO_3_). It is considered as generally recognized as safe by the FDA and is widely used as a food additive and also in various oral drugs. Due to its low cost, ease of manufacture, and biodegradability, CaCO_3_ particles have been studied in vitro as potential carriers for various drugs^22^, ^23^, ^24^, ^25^, ^26^, ^27^ and in monkeys and humans for intranasal administration of insulin.^28^

Internal radiation therapy with radiolabeled particles has been a treatment option for cancers with intracavitary dissemination, eg, for patients with peritoneal carcinomatosis from ovarian carcinoma.^29^ This condition occurs when cancer cells from a primary tumor in an adjacent organ disseminate into the peritoneal cavity and cause micrometastases by adhering to the serosal surfaces of the peritoneal lining. Peritoneal metastases are the most common terminal feature of abdominal cancers and generally imply a poor prognosis for the patients affected.^30^ Intraperitoneal (IP) radionuclide therapy has been evaluated in clinical trials with suspensions of radioactive nanoparticles or microparticles^31^, ^32^ and, more recently, also as radioimmunotherapy.^33^, ^34^, ^35^, ^36^, ^37^ An overview of the physical characteristics of the different radionuclides that have been utilized is given in Table 1. With particles as carriers, only the β‐emitters ^198^Au and ^32^P have been examined in patients. Of the 2, IP therapy with ^32^P‐colloid was the most successful.^32^, ^38^ In a randomized trial, it was reported to be as effective as adjuvant cisplatin for treatment of ovarian cancer, although late bowel complications occurred more frequently.^32^ This complication was also seen after ^198^Au‐colloid therapy.^29^ One possible cause of the adverse effects is the emitted electrons, which have a range of several millimeters in tissue and therefore could irradiate deeper areas of radiation sensitive organs in the abdomen, like the small intestine.

**Table 1:** Physical characteristics of the radionuclides previously investigated clinically for local intraperitoneal therapy compared with ^224^Ra

Radionuclide	Half‐Life	Decay Mode(s)	Energy^a^ (MeV/Bq‐s)	Carrier
^32^P	14.3 days	β	0.695	Particles^32^
^90^Y	2.7 days	β	0.934	Antibody^33^
^131^I	8.0 days	β	0.573	Antibody^34^
^177^Lu	6.7 days	β	0.131	Antibody^35^
^198^Au	2.7 days	β	0.731	Particles^31^
^211^At	7.2 h	α	6.96	Antibody fragment^36^
^212^Pb	10.6 h	β + α	10.27	Antibody^37^
^224^Ra	3.6 days	4α + 2β	29.26	n/a

a From ENSDF decay data in MIRD format (http://www.nndc.bnl.gov/mird). Listed α, β, γ, and X‐rays for mother nuclides and, if applicable, progeny combined.

Our long‐term goal is to develop a novel radium compound for locoregional treatment of cancers in cavitary regions. By utilizing the α‐emitting ^224^Ra, with a penetration depth in tissue of less than 0.1 mm, the aim is to design a radiotherapeutic microparticle for intracavitary injection with highly localized effect on cancer cells residing on serosal surfaces, with minimal normal organ exposure. Microparticles were chosen as carriers for ^224^Ra, as it is possible to select a size that facilitates high retention of the particles in the peritoneal cavity.^39^, ^40^ Consequently, the radiolabeled microparticles will irradiate the area where the peritoneal micrometastases are located. In this study, we have investigated the suitability of CaCO_3_ microparticles as carriers for ^224^Ra. The complete preparation of the product is described, and the biodistribution in mice after IP injection of the radiolabeled microparticles is evaluated.

## 2 EXPERIMENTAL

### 2.1 Calcium carbonate microparticles

Crystalline CaCO_3_ microparticles were prepared by a spontaneous precipitation method based on the protocol described by Volodkin et al.^41^ Five milligrams of 0.33 M Na_2_CO_3_ (Merck, Darmstadt, Germany) solution was rapidly poured into an equal volume of 0.33 M CaCl_2_ (Merck). First‐generation microparticles were prepared by intense vortexing of the mixture for 30 seconds before the particle suspension was left for 5 minutes. The precipitate was collected by using filtration through a 0.45‐ μm nitrocellulose filter (Whatman, GE Healthcare, UK) in a glass vacuum filtration device, before it was washed with approximately 30‐ mL ph. Eur water (complying with quality standards of the European Pharmacopeia, VWR, Oslo, Norway) and dried overnight at room temperature. Second‐generation microparticles were prepared by mixing of 0.33 M Na_2_CO_3_ and CaCl_2_ solutions with a magnetic stirrer (Biosan MS3000, Riga, Latvia) before the precipitated particles were collected by centrifugation. The precipitate was washed in ph. Eur water and dried for 1 hour at 180°C (VENTI‐Line Drying oven VL53, VWR). In addition, a batch of CaCO_3_ microparticles was purchased from PlasmaChem GmbH (Berlin, Germany). A portion of the microparticles were dry sterilized at 180°C for 2 hours. The 3 different particle types were analyzed by laser diffraction in a Mastersizer 3000 (Malvern Instruments Ltd, Worcestershire, UK), and volume‐based diameters were obtained. The microparticles were also analyzed for visualization of crystal shape, size, and surface morphology with scanning electron microscopy (SEM) performed at Particle Analytical (Hørsholm, Denmark) with a Leica Stereoscan 360.

### 2.2 Preparation of ^224^Ra generator based on ^228^Th

The ^224^Ra generator was prepared by mixing a ^228^Th source with an actinide resin and loading it on a column. A source of ^228^Th in 1 M HNO_3_ was purchased from Eckert & Ziegler (Braunschweig, Germany), and an actinide resin based on the DIPEX^®^ Extractant was acquired from Eichrom Technologies LLC (Lisle, IL) in the form of a pre‐packed cartridge of 2 mL. The material in an actinide resin cartridge was extracted, and the resin was preconditioned with 1 M HCl (Sigma‐Aldrich). A slurry of approximately 0.25‐ mL actinide resin, 0.25‐ mL 1 M HCl, and 0.1‐ mL ^228^Th in 1 M HNO_3_ was prepared in a vial (4‐mL vial, E‐C sample, Wheaton, Millville, NJ) and incubated with gentle agitation for immobilization of ^228^Th for 4 hours at room temperature and let to rest for a few days. The generator column was prepared in a 1‐ mL filtration column (Isolute SPE, Biotage AB, Uppsala, Sweden) by first applying 0.2 mL of inactive actinide resin, before the portion containing ^228^Th was loaded on top. The inactive resin was introduced in the bottom of the column to serve as a catcher layer if ^228^Th was released during operation of the generator. Later, the capacity of the generator was increased. A slurry consisting of 0.4‐ mL actinide resin, 0.5‐ mL ^228^Th in 1 M HNO_3_, and 0.5‐ mL 1 M HCl was prepared as described above, before it was loaded onto the generator column. At its maximum capacity, the ^224^Ra generator column contained approximately 2‐ MBq ^228^Th.

### 2.3 Extraction of ^224^Ra

Radium‐224 could be eluted regularly from the generator column in 1 to 2 mL of 1 M HCl. For further purification, the crude eluate from the generator column was loaded directly onto a second actinide resin column. The second column was washed with 1 M HCl. This eluate was evaporated to dryness in a closed system. The vial was placed in a heater block (heated to approximately 100°C) and flushed with N_2_ gas through a Teflon tube inlet and outlet in the rubber/Teflon septum on the vial. The acid vapor was led into a beaker of saturated NaOH by a stream of N_2_ gas. The radioactive residue remaining after evaporation was dissolved in 0.2 mL or more of 0.1 M HCl. A radioisotope calibrator (CRC‐25R, Capintec Inc., Ramsey, NJ) was used to measure the total extracted activity in the process. Possible breakthrough of ^228^Th in the final ^224^Ra solution was examined by sending 2 samples from different eluates for analysis at Institute for Energy Technology (Kjeller, Norway). In 1 sample, the ^224^Ra and ^228^Th content was determined by radiochemical separation followed by α‐spectroscopy. To detect possible ingrowth of ^224^Ra from ^228^Th, the second sample was measured repeatedly over a period of 40 days with liquid scintillation to determine the total amount of α‐emitters.

### 2.4 Radioactivity measurements

Radioactive samples were measured in the window 70 to 80 keV on a Cobra II Autogamma counter (Packard Instruments, Downer Grove, IL) or from 65 to 345 keV on a Hidex Automatic Gamma Counter (Hidex, Turku, Finland). From 70 to 80 and 65 to 345 keV, the most abundant X‐ and γ‐radiation is from the ^224^Ra daughter ^212^Pb, and it is therefore assumed that the counts in these windows mainly originate from ^212^Pb with minimal contribution from other nuclides in the series. This can be seen from Table 2, which provides an overview of X‐ and γ‐rays from the different nuclides in the ^224^Ra series. Because ^224^Ra decay results in modest γ‐emission in an energy region with more abundant γ from its progeny ^212^Pb, the ^224^Ra activity was determined indirectly based on the counts in the 70 to 80‐ keV or 65 to 345‐ keV window. This was carried out by re‐measuring the samples between 1 and 4 days after the first measurement, when the initial ^212^Pb present in the sample had decayed and equilibrium between ^224^Ra and newly produced ^212^Pb had been established. A pure source of ^224^Ra reaches equilibrium conditions after approximately 2 days.

**Table 2:** Overview of X‐ and/or γ‐lines in the ^224^Ra series with 1% or higher abundance

Nuclide	65‐345 keV (Abundance)		>345 keV (Abundance)	
^224^Ra	241.0 keV	(4.1%)		
^220^Rn				
^216^Po				
^212^Pb	74.8 keV	(10.3%)		
	77.1 keV	(17.1%)		
	86.8 keV	(2.1%)		
	87.4 keV	(4.0%)		
	89.8 keV	(1.5%)		
	238.6 keV	(43.6%)		
	300.1 keV	(3.3%)		
^212^Bi			727.3 keV	(4.3%)^a^
^212^Po				
^208^Tl			510.8 keV	(8.1%)^a^
	75.0 keV	(1.2%)^a^	583.2 keV	(30.5%)^a^
	277.4 keV	(2.4%)^a^	860.6 keV	(4.5%)^a^
			2614.5 keV	(35.8%)^a^

The X and γ‐lines are divided into 2 columns, 1 for energies between 65 and 345 keV and the other for energies larger than 345 keV. The energies and abundance were retrieved from http://www.nndc.bnl.gov/chart.

Branching corrected for 64.1%.

a Branching corrected for 35.9%.

### 2.5 Labeling of CaCO_3_ microparticles with ^224^Ra

Radium‐224‐labeled microparticles were prepared by precipitation of ^224^Ra^2+^ on the CaCO_3_ surfaces. From 10 to 200 mg of CaCO_3_ microparticles were transferred to an Eppendorf tube and washed 3 times in 1‐ mL water and 2 times with 1‐ mL 0.1 M Na_2_SO_4_ (Alfa Aesar, Karlsruhe, Germany). The particles were separated from the washing solution by centrifugation (2000×*g* for 30‐180 s, Spectrafuge^™^ Mini, Labnet Inc.). After wash, the microparticles were dispersed in either Dulbecco's phosphate‐buffered saline (PBS; Gibco, Life Technologies, Carlsbad, CA) supplemented with 0.5% bovine serum albumin (BSA), a sucrose solution, or 0.9% NaCl (Merck). The sucrose solution contained 94‐ mg/mL sucrose (Sigma Ultra, St. Louis, MO) and 2.1‐mg/mL Na_2_SO_4_ (Alfa Aesar) and was pH adjusted to 7.5. The use of a sucrose solution for radiolabeling of the microparticles was explored because of the viscosity of the solution. In the case of using Dulbecco's PBS with 0.5% BSA, the particle suspension was incubated with orbital rotation on a HulaMixer (Invitrogen, Life Technologies, Carlsbad, CA) for 30 minutes at room temperature before continuing the protocol. A solution of ^224^Ra in equilibrium with progeny in 0.1 M HCl and 0.5 M NH_4_OAc (Merck), and pH between 5 and 6 was added to the particles together with small amounts of SO_4_^2−^ and Ba^2+^ used as co‐precipitants. The volumes of 0.1 M Na_2_SO_4_ and 0.07 M BaCl_2_·2H_2_O (Merck) solutions corresponded to 0.3% of SO_4_^2−^ and Ba^2+^ per mg of particles, respectively. Microparticles in radiolabeling solution were incubated with orbital rotation on a HulaMixer for minimum 90 minutes at room temperature. After incubation, the particle solution was washed twice with 1 mL of either sucrose solution or 0.9% NaCl, and the wash solutions collected in separate tubes. The radioactivity (counts per minute, CPM) in the particle suspension (P) and washing solutions (W1 and W2) was measured, and the ^212^Pb labeling efficiency was estimated as the percentage of the total activity still bound to the microparticles after the labeling procedure:

$$\%\text{Labeling efficiency}=\frac{\mathrm{CPM}\left(P\right)}{\mathrm{CPM}\left(P+W1+W2\right)}\times 100$$

All samples were left to decay for minimum 24 hours at room temperature, to reach equilibrium between ^224^Ra and ^212^Pb, before they were re‐measured, and the ^224^Ra labeling efficiency was calculated with the same equation as presented above. It is assumed here that equilibrium between ^224^Ra and ^212^Pb in the samples is reached after 24 hours because the samples are expected to have a relatively even distribution of the 2 nuclides; ie, equilibrium will be reached faster than from a pure source of ^224^Ra.

### 2.6 In vitro stability of ^224^Ra‐labeled CaCO_3_ microparticles

To determine the retention of ^224^Ra and ^212^Pb on the microparticles after labeling, the particles were incubated in 1‐ mL sucrose solution at room temperature. After 1, 3, 5, and 7 days, the particle suspension was centrifuged and 80% of the supernatant was withdrawn into a separate vial. Afterward, if the stability study was to be continued to a later time point, the particle pellet was dispersed in a new aliquot of sucrose solution and incubated further. The radioactivity in the removed supernatant (S) and particle suspension (P) was measured, and the estimated activity on the particles without supernatant was divided by the absolute amount of activity in the sample to determine the fraction of ^212^Pb retained on the particles:

$$\%\text{Retained activity}=\frac{\mathrm{CPM}\left(P\right)-\left(\mathrm{CPM}\left(S\right)\;\times \;\frac{20}{80}\right)}{\mathrm{CPM}\left(P+S\right)}\;\times \;100.$$

Two days after the first measurement, when equilibrium between ^224^Ra and ^212^Pb had been reached, the samples were re‐measured and the retained ^224^Ra activity was estimated with the equation given above.

### 2.7 Biodistribution and in vivo stability of ^224^Ra‐labeled CaCO_3_ microparticles

Institutionally bred, 4 to 48 weeks old, healthy female Athymic nude Foxn^nu^ mice with body weights in the range of 17.1 to 28.5 g at the start of the experiment were used. The animals were maintained under pathogen‐free conditions with food and water supplied ad libitum. All procedures and experiments involving animals were approved by the National Animal Research Authority (permit ID 6675) and performed in compliance with regulations set by the same authority and the EU Directive 2010/63/EU on the protection of animals used for scientific purposes.

The biodistribution of radioactivity in mice was studied after a single IP injection of 0.4‐mL ^224^Ra‐labeled CaCO_3_ microparticle suspension. Table 3 gives an overview of the different biodistribution experiments performed with ^224^Ra‐labeled CaCO_3_ microparticles. Biodistribution experiments with free ^224^Ra (dissolved RaCl_2_) were also performed, by administering approximately 3 kBq ^224^Ra in 0.25 mL of 0.9% NaCl solution with pH of 5.5 IP to each mouse. Groups of 2 to 5 mice were sacrificed 1, 4, or 7 days post injection. Blood was collected by cardiac puncture, while the mice were under sevoflurane anesthesia. Immediately after blood sampling, the animals were euthanized by cervical dislocation before lungs, heart, liver, spleen, kidney, stomach, intestines, femur, muscle, brain, scull, diaphragm, parietal peritoneum, and IP fat from the lower abdomen (described by Clark et al^42^ as uterine fat) were harvested. Each collected sample was weighed and the radioactivity measured in a γ‐counter to determine the activity per gram tissue. Measurements of the samples approximately 3 hours after the mice were sacrificed were used to estimate the amount of ^212^Pb, whereas re‐measurements of the samples 3 to 4 days after euthanization were used to determine the amount of ^224^Ra. The data for ^224^Ra were decay corrected to estimate the activity in the sample at time of sacrifice. Samples of the injectates were used to estimate the actual injected dose per mouse before the injected dose was normalized to 10 kBq per mouse.

**Table 3:** Overview of biodistribution experiments with ^224^Ra‐labeled CaCO_3_ microparticles

Particle Batch	Labeling Solution	Injection Solution	Injected Activity	Particle Dose	Time Points	Group Size
First generation	DPBS with 0.5% BSA	Sucrose solution	6‐9 kBq		1 day	5
			2 kBq	5 mg	4 days	3
			2 kBq		7 days	3
First generation	Sucrose solution	Sucrose solution	12 kBq	5 mg	1 day	3
PlasmaChem	Sucrose solution	Sucrose solution	16 kBq	5 mg	1 day	3
Second generation	0.9% NaCl	0.9% NaCl	16 kBq	1 mg	1 day	2
			16 kBq	5 mg	1 day	3
			22 kBq	25 mg	1 day	3

## 3 RESULTS

### 3.1 Characterization of CaCO_3_ microparticles

**Figure 2:**
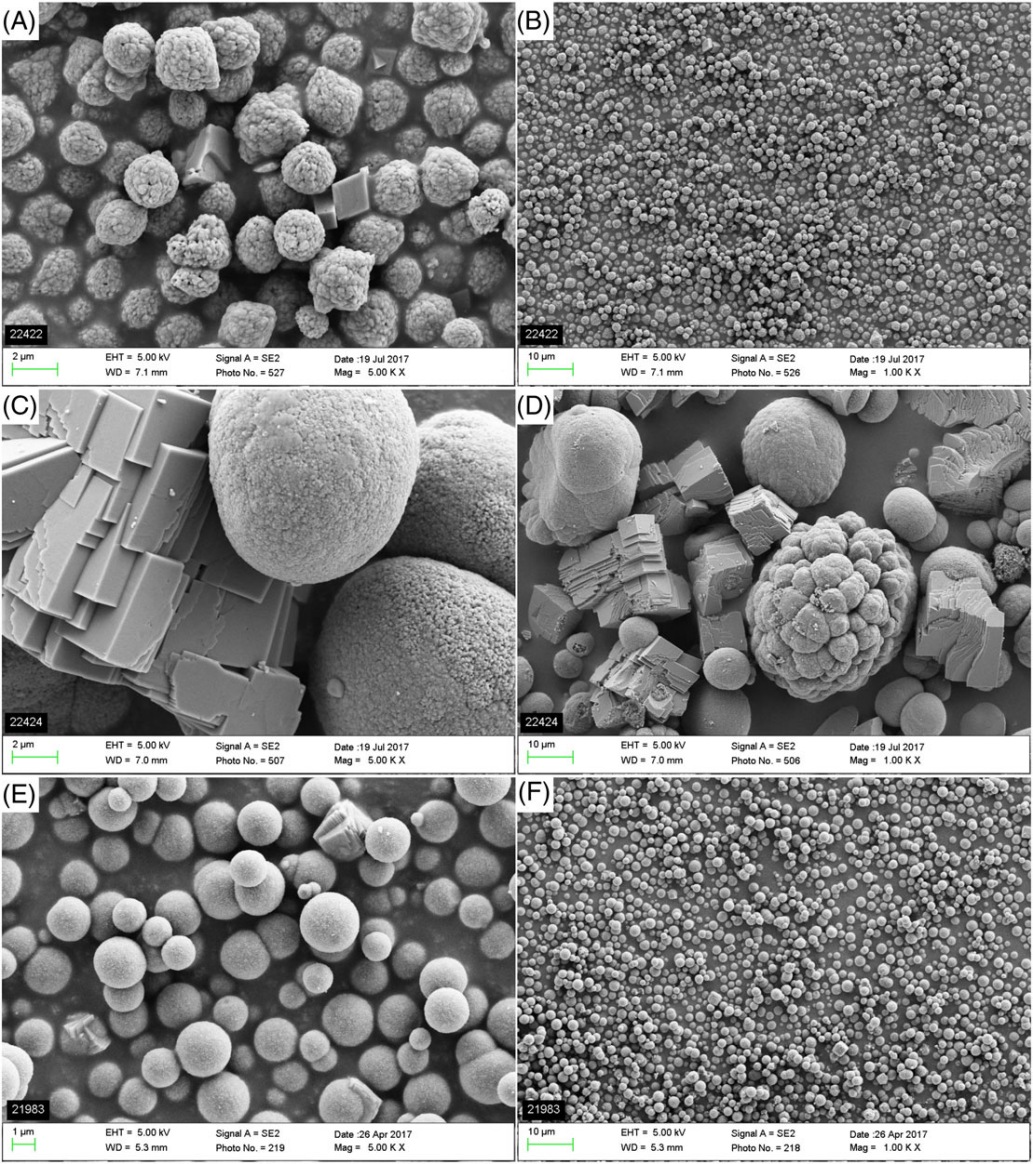
Scanning electron microscopy images showing the 3 CaCO_3_ microparticle batches used in this study: (A and B) PlasmaChem, (C and D) first generation, and (E and F) second generation

Characterization of the CaCO_3_ microparticles was performed by determining the size distribution with laser diffraction, and individual particle diameters, crystal shape, and surface morphology were visualized with SEM imaging. The size distribution was described by the D10, D50, and D90 values, which represent the diameters where 10, 50, and 90% of the population lie below. These numbers are presented in Table 4 together with a summary of observations from SEM images displayed in Figure 2. PlasmaChem and the second‐generation microparticles produced by us were spherical and relatively uniform, with individual particle sizes from 1 to 3 μm in diameter. The first‐generation microparticles consisted of both spherical and rhombohedral crystals. In general, this particle population was more heterogeneous, with individual particle diameters ranging from approximately 3 to 15 μm. The surface morphology of the spherical particles is smoother for the first‐generation and second‐generation particles, whereas the surface of the PlasmaChem particles appears slightly rougher and more “raspberry‐like.” The relatively higher D90 value obtained with laser diffraction compared with individual particle sizes observed in the SEM images is probably caused by clusters of particles being measured as one unit.

**Table 4:** Characteristics of the 3 CaCO_3_ microparticle batches used in this study

Particle Batch	Volume‐Based Diameters (μm)			Individual Particle Shape
	D10	D50	D90	
PlasmaChem	2.4 ± 0.1	5.1 ± 0.3	10.5 ± 0.5	Spherical
First generation	9.4 ± 0.2	17.6 ± 0.9	31.4 ± 2.6	Spherical and rhombohedral crystals
Second generation	3.0 ± 0.4	7.0 ± 1.1	15.3 ± 3.1	Spherical

### 3.2 Ra‐224 generator performance

Breakthrough of the ^228^Th parent was determined with α‐spectroscopy to be less than or equal to 1.5 × 10^−3^ Bq/mL. This amount corresponds to less than 3 × 10^−7^ of the original ^224^Ra activity. No ingrowth of ^224^Ra from ^228^Th was detected when half‐life measurements with liquid scintillation were performed. Altogether, the results from these 2 analyses suggest that the quality of the prepared ^224^Ra solution was satisfactory.

### 3.3 Radiolabeling of CaCO_3_ microparticles

Radiolabeling of CaCO_3_ microparticles was successful, and the results presented in Table 5 showed that both ^224^Ra and daughter nuclide ^212^Pb were adsorbed with yields above 80% by the microparticles, regardless of differences in median particle size and composition of the labeling solution. The method is based on precipitation of ^224^Ra^2+^ and ^212^Pb^2+^ together with Ba^2+^ as sulfates onto the microparticle surfaces because of the low solubility of these inorganic salts.^43^, ^44^ The highest labeling efficiencies were obtained when the second‐generation microparticles were radiolabeled by using 0.9% NaCl as the main component in the labeling solution. With this combination, average labeling efficiencies above 95% for both ^224^Ra and ^212^Pb were achieved. When comparing PlasmaChem and the first‐generation microparticles, which were radiolabeled with the same solutions but had significantly different median size, there was a tendency toward higher labeling efficiency of ^212^Pb and ^224^Ra for the smallest particles. This might be caused by a relatively higher total surface area of the smaller particles compared with the larger. No significant differences in labeling efficiencies were observed when BSA was added to the labeling solution.

**Table 5:** The labeling efficiencies (±standard deviation) of ^224^Ra and daughter ^212^Pb for different CaCO_3_ microparticles

Particle Batch	Labeling Solution	Labeling Efficiency		n
		Pb‐212	Ra‐224	
PlasmaChem	Sucrose solution	89.7 ± 7.5	94.2 ± 4.9	21
	DPBS with 0.5% BSA	93.6 ± 4.0	87.5 ± 7.3	3
First generation	Sucrose solution	82.7 ± 10.7	84.8 ± 9.6	18
	DPBS with 0.5% BSA	86.4 ± 6.7	82.2 ± 10.0	3
Second generation	0.9% NaCl	96.5 ± 2.8	96.6 ± 1.9	8

The results are showed for sucrose solution. Dulbecco's phosphate‐buffered saline (DPBS) added 0.5% bovine serum albumin (BSA) and 0.9% NaCl as the main component in the labeling solution. Number of independent labeling experiments is given in the last column denoted with n.

### 3.4 Retained activity on CaCO_3_ microparticles in vitro

**Figure 3:**
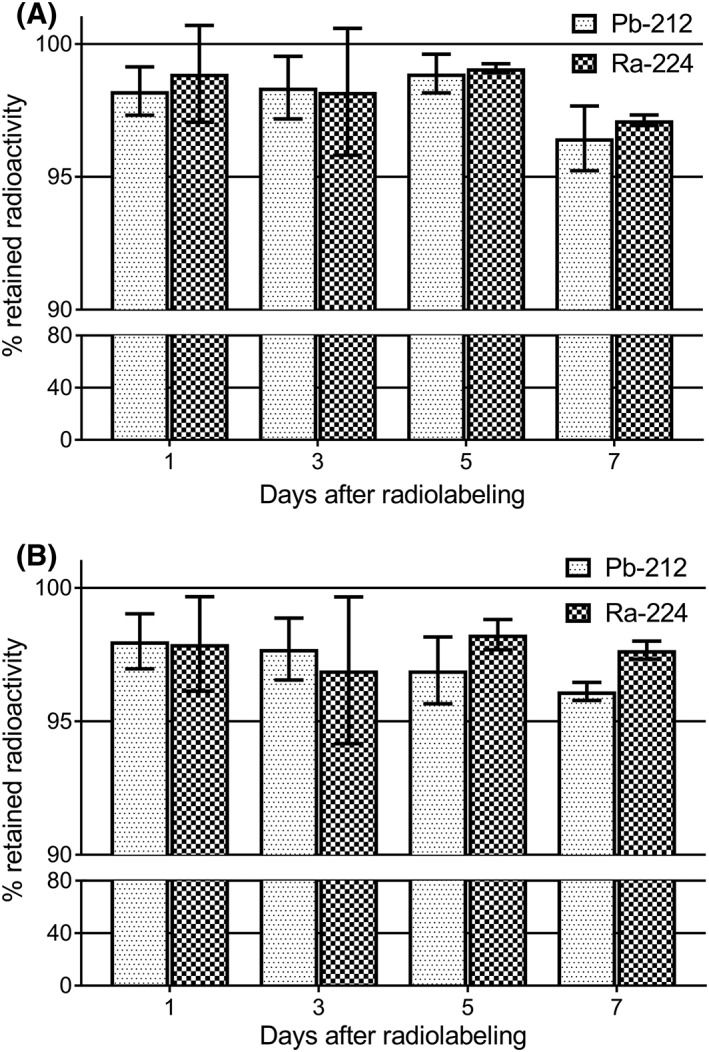
Bar graph illustrating the retained ^224^Ra and daughter ^212^Pb activity on CaCO_3_ microparticles stored in sucrose solution, of median size 5 μm (A, PlasmaChem) and 18 μm (B, first generation) at different time points after labeling in sucrose solution. The bars represent the mean value of 4 to 14 experiments, and the error bars represent the standard deviation

The ability of the CaCO_3_ microparticles to retain the adsorbed radionuclides was examined in vitro to evaluate whether a concentrated solution of particles was stable in a relevant formulation for in vivo injection within the time frame of the ^224^Ra half‐life. It was found that microparticles ^224^Ra‐labeled in sucrose solution retained most of the adsorbed activity when stored in the same solution in a period up to 7 days. Figure 3 shows that the average retained activity of ^224^Ra and daughter ^212^Pb was above 95% for all time points for microparticles of 2 different sizes, the PlasmaChem and the first generation particles produced by us. Because most of the supernatant (80%) was removed and replaced with new solution at every time point, the data probably reflect the solubility of the CaCO_3_ microparticles in solution. This is likely as there were no significant differences in retained activity between the different time points. It must be emphasized that the retention experiments described here were not intended to mimic in vivo conditions, but rather to determine if the radiolabeled CaCO_3_ microparticles had a sufficient stability in sucrose solution within a period which is compatible with the half‐life of ^224^Ra.

### 3.5 Biodistribution and in vivo stability of ^224^Ra‐labeled CaCO_3_ microparticles

**Figure 4:**
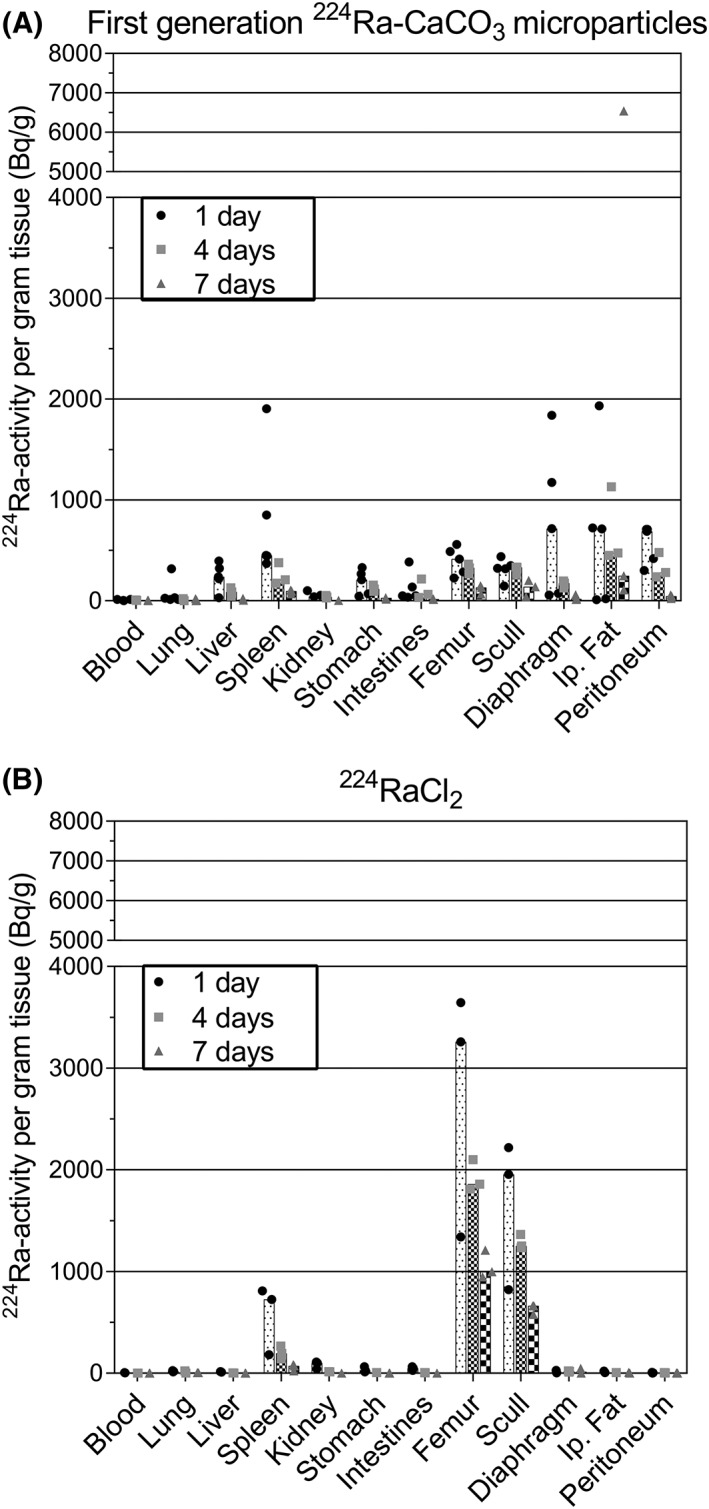
Biodistribution 1, 4, and 7 days following intraperitoneal injection of ^224^Ra‐labeled first‐generation CaCO_3_ microparticles (A) and dissolved ^224^RaCl_2_ (B) in athymic nude mice. The particles were labeled in Dulbecco's phosphate‐buffered saline with 0.5% bovine serum albumin and administered in sucrose solution. The data are shown as a bar representing the median ^224^Ra activity per mass unit in addition to the individual data points for each mouse. The injected activity has been normalized to 10 kBq per mouse

Results from the biodistribution studies showed that the radioactivity was mainly located in the peritoneal area after administration of the first generation CaCO_3_ microparticles labeled with ^224^Ra in Dulbecco's PBS with 0.5% BSA and administered in sucrose solution. In Figure 4, the tissue distribution 1, 4, and 7 days after injection is displayed as radioactivity per gram tissue for the radiolabeled microparticles compared with free ^224^Ra. After administration of ^224^Ra‐labeled CaCO_3_ microparticles, activity was measured for all IP organs and tissues, with the highest activity associated with the IP fat and on the parietal peritoneum and diaphragm. On the other hand, when free ^224^Ra was administered, the spleen was the only IP organ where a noteworthy activity level was observed. The measured activity in spleen after free ^224^Ra injection is likely due to ^224^Ra released to the blood stream which has redistributed, as some calcification occurs in the spleen of mice.

Because of the bone‐seeking property of ^224^Ra, the retention of ^224^Ra on the radiolabeled particles in vivo could be examined by comparing the skeletal uptake of ^224^Ra with that after IP administration of free ^224^Ra. After injection of the first‐generation microparticles, the femur and scull uptake was about one sixth or less compared with the activity in the skeleton of mice given free ^224^Ra. Based on these data, it is seen that some release of ^224^Ra from the microparticles occurs, but it is relatively modest. Among the other nonabdominal organs and tissues examined, no shift in tissue radiation exposure was observed when ^224^Ra‐labeled CaCO_3_‐microparticles were administered. The radioactivity in heart, muscle, and brain was lower than 100 Bq/g for both free ^224^Ra and ^224^Ra‐labeled CaCO_3_ microparticles.

The biodistribution of the ^224^Ra daughter with the longest half‐life, ^212^Pb, was also measured after IP administration of first generation ^224^Ra‐labeled CaCO_3_ microparticles and free ^224^Ra (data shown in Figure S1). The main difference in tissue distribution of these 2 nuclides was the higher measured ^212^Pb activity in kidneys. After injection of the radiolabeled microparticles, the measured ^212^Pb activity in kidneys was higher than for mice given free ^224^Ra. The ratio of ^212^Pb in kidneys when ^224^Ra was bound to the microparticles to the average uptake in kidneys when ^224^Ra was given alone ranged from 1.4 to 2.1 at the different time points. This suggests that a portion of the measured activity originates from ^212^Pb that had redistributed to the kidneys via the circulation system and the other fraction from radiolabeled microparticles located on the kidney surface.

#### 3.5.1 Effect of microparticle size

**Figure 5:**
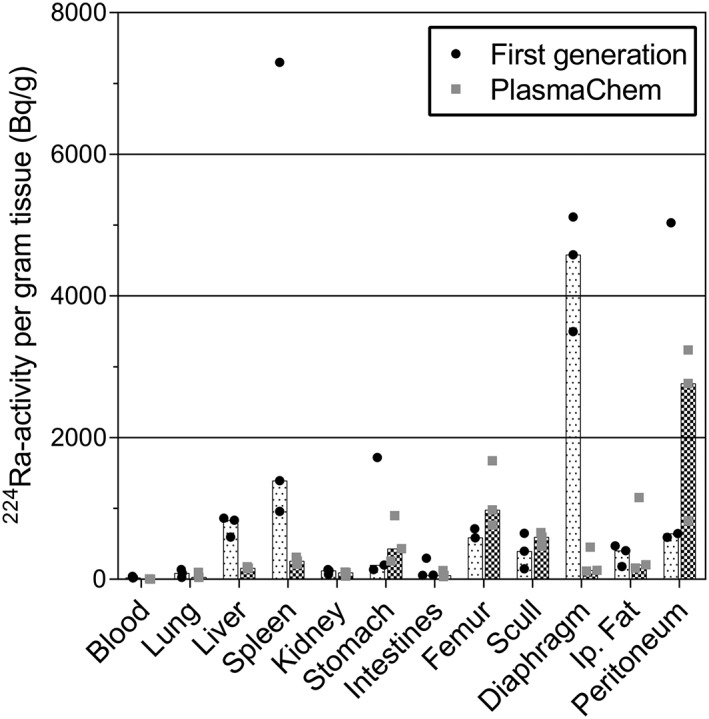
Comparison of biodistribution 1 day after intraperitoneal injection of ^224^Ra‐labeled CaCO_3_ microparticles with small (PlasmaChem) and large (first generation) median diameter in athymic nude mice. The particles were labeled and administered in sucrose solution. The data are shown as a bar representing the median ^224^Ra activity per mass unit in addition to the individual data points for each mouse. The injected activity has been normalized to 10 kBq per mouse

A comparison of the biodistribution 1 day after administration of ^224^Ra‐labeled CaCO_3_ microparticles of 2 different sizes, first generation and PlasmaChem, both labeled in sucrose solution, was performed. The results are presented in Figure 5 as the radioactivity per gram tissue. A few differences between the 2 particle sizes were seen. The release of ^224^Ra from the microparticles, measured indirectly by the skeletal uptake, was found to be slightly higher with the smallest particles. For the small particles, the skeletal uptake was about one third of the activity found when the mice were administered free ^224^Ra IP (Figure 4B), compared with approximately one fifth in the larger first generation particles. The IP tissues with the highest measured activity were the diaphragm for the large particles and the peritoneum for the small type. Because these tissues also exhibited some degree of intragroup variation in the data presented in Figure 4A, this difference might be random. In general, the data obtained are comparable with what was found with the first‐generation microparticles labeled with Dulbecco's PBS with 0.5% BSA (Figure 4A), apart from a notably higher measured activity in the liver and spleen with the first‐generation particles labeled in sucrose solution. Because the high activity in these organs was not observed with PlasmaChem and to a lesser extent when the first‐generation microparticles were labeled by using BSA, it is not likely to be a consequence of the absence of BSA in the labeling solution. One possible explanation for the variability in the measured activity in the spleen can be incomplete removal of the surrounding fat. Generally, high activity levels were measured in the IP fat after administration of the radiolabeled microparticles. The presence of fat associated with the spleen could therefore greatly influence the measured activity and cause the high levels.

#### 3.5.2 Effect of amount of microparticles

**Figure 6:**
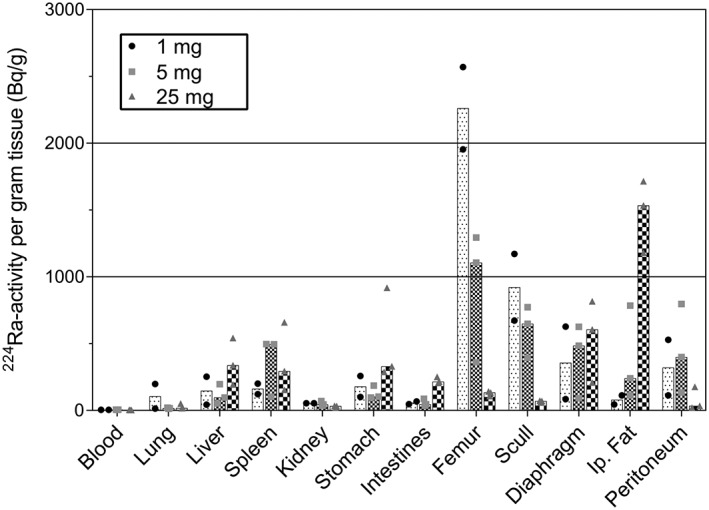
Biodistribution in athymic nude mice 1 day following intraperitoneal administration of 1, 5, and 25 mg of ^224^Ra‐labeled second generation CaCO_3_ microparticles. The particles were labeled and administered in 0.9% NaCl. The data are shown as a bar representing the median ^224^Ra activity per mass unit in addition to the individual data points for each mouse. The injected activity has been normalized to 10 kBq per mouse

The effect of amount of carrier was investigated by comparing the biodistribution 1 day after administration of 1, 5, and 25 mg of second‐generation microparticles ^224^Ra‐labeled and administered in 0.9% NaCl. The results are mainly similar to the previously presented biodistribution results, but with 1 striking difference. It is seen in Figure 6 that the skeletal uptake, and thereby also the ^224^Ra release, is strongly correlated to the amount of microparticles administered. The level of ^224^Ra in the femurs of mice given 1‐ mg ^224^Ra‐labeled CaCO_3_ microparticles was only 30% less than after IP injection of free ^224^Ra, showing a very high release. On the contrary, the release was almost negligible after injection of 25‐ mg ^224^Ra‐labeled CaCO_3_ microparticles. The skeletal uptake was only 4% of what was found in the skeleton of mice 1 day after administration of free ^224^Ra. The intermediate amount, 5 mg, also exhibited intermediate stability with almost identical skeletal uptake after 1 day as when 5‐ mg PlasmaChem microparticles labeled and administered in sucrose solution were used. This observation is consistent with the PlasmaChem and second‐generation particles being of the approximate same size, as the previous results showed that ^224^Ra release from the particles was somewhat dependent on size.

#### 3.5.3 Microparticle distribution in the peritoneal cavity

In most of the mice, particulate residue was observed at necropsy. The residue was found regardless of the microparticle type and amount of particles and also independent of the different labeling and injection solutions that were used. The amount of residue increased with increasing amount of particles administered. When 1 and 5‐ mg particles were administered, the residue was relatively small, below 3 mm in diameter, and was always found on the anterior parietal peritoneum. After administration of 25‐ mg particles, the residue was larger and several pieces were observed. It was also always located on the anterior parietal peritoneum but in addition found more spread out in the entire peritoneal cavity. All observed residues were either completely free‐floating or loosely associated with an adjacent abdominal structure and thus were not collected for measurements.

The points in all biodistribution graphs show the individual values for each mouse together with a bar representing the median activity per mass unit. The median was chosen to be more representative for the central tendency of the data than the mean, as in some IP organs and tissues there were significant intragroup variation after administration of the radiolabeled microparticles. The variation may be caused by a heterogeneous particle distribution in the peritoneal cavity or particle aggregates.

## 4 DISCUSSION

Radium‐224 is a radionuclide with unrealized therapeutic potential. Despite its attractive properties, it is often overlooked when candidates for α‐therapy are mentioned,^1^, ^45^ probably because of lack of a stable chelator for in vivo use in targeted therapies. In the current study, we have proposed CaCO_3_ microparticles as carriers for ^224^Ra, specifically designed for local treatment of cancer in cavitary regions, such as the peritoneal cavity. We have demonstrated that preparation of the ^224^Ra‐labeled CaCO_3_ microparticles by a simple and efficient procedure resulted in high labeling efficiencies. The in vitro stability of the radiolabeled microparticles showed that the radionuclides were retained on the particles to a high degree for at least 1 week. This time frame should be sufficient for production and distribution of the ^224^Ra‐labeled CaCO_3_ microparticles to the end user and be compatible with the half‐life of ^224^Ra.

The biodistribution of the ^224^Ra‐labeled CaCO_3_ microparticles in mice after IP administration was encouraging, with high IP retention of radioactivity. When 5 mg or more of the radiolabeled microparticles were administered, only a limited amount of ^224^Ra was systemically distributed. The measured radioactivity in abdominal organs and tissues after injection of radiolabeled microparticles was therefore assumed to mainly originate from exposure to serosal surfaces and not from ^224^Ra relocalized to IP organs via the circulation, as the biodistribution of free ^224^Ra did not show uptake in any IP organs, except from the spleen. Exposure to serosal surfaces is a prerequisite for the ^224^Ra‐labeled CaCO_3_ microparticles to have therapeutic effect, as this is where the peritoneal micrometastases are located. Particles can be cleared from the peritoneal cavity either by lymphatic drainage or by interactions with phagocytic resident cells, like peritoneal macrophages. Several studies have shown a correlation between particles size and clearance from the peritoneal cavity.^39^, ^46^, ^47^ Although different particle types have been examined, it is, in general, supported that particles with diameters above 1 μm are retained to a high degree in the peritoneal cavity, whereas smaller are more rapidly cleared. Altogether, the biodistribution results indicate that the size range of microparticles examined here primarily remain IP, which has also been shown in the literature,^39^, ^40^, ^46^, ^47^ and thereby supports our hypothesis that the ^224^Ra‐labeled microparticles may be effective in irradiation of liquid volumes and serosal surfaces in the peritoneal cavity when administered in a suitable dose. Another interesting finding was the substantial amount of radioactivity associated with the IP fat. It is known that several types of IP cancers have a clear preference to metastasize in IP adipose tissues, and especially in the omentum.^48^, ^49^ The accumulation of radioactivity in the IP fat may therefore be advantageous in a clinical setting.

It was seen from the biodistribution studies that there was some release of both ^224^Ra and daughter ^212^Pb from the microparticles in vivo. Usually, it is viewed as negative to have some release of radionuclides from a product. That is especially the case when a radionuclide with multiple α‐emitting daughters, such as ^224^Ra, is involved. A poor retention at the target site can cause distant toxicity due to an unfavorable redistribution of progeny in the body. However, both the acute and long‐term toxicity, as well as tolerability of ^224^Ra, are well documented after several years of clinical use for treatment of ankylosing spondylitis. The ^224^Ra that is released from the microparticles and into the circulation accumulates rapidly in bone and the highest risk of unwanted effects is therefore bone marrow toxicity. In the treatment of ankylosing spondylitis, weekly injections of 1‐ MBq ^224^Ra‐dichloride were given intravenously, up to a total of 10 injections. Reports including almost 1000 patients receiving this treatment regimen have shown that this dosing could be administered without significant bone marrow toxicity.^6^, ^50^, ^51^ These data indicate that a modest level of ^224^Ra release from the microparticles can be acceptable, but an assessment of marrow toxicity should be included in future studies.

An interesting discovery was the strong dependence of ^224^Ra release on the amount of CaCO_3_ microparticles administered. With this property, it is possible both to have almost all of the radioactivity remaining in the peritoneal cavity by giving a high microparticle dose or reducing the dose to have some ^224^Ra release. The differences in stability between the doses probably reflect the solubility of the CaCO_3_ microparticles. When more microparticles are present, a smaller percentage of the particles are needed to obtain the solubility equilibrium of Ca^2+^ and CO_3_^2−^, and thus, less ^224^Ra is released.

A potential downside with therapeutic microparticle suspensions can be a heterogeneous distribution in the peritoneal cavity. If the microparticles do not disperse evenly in the peritoneal cavity, some spots might have more particles and some less, which, in worst case, can lead to dose inhomogeneity and tumor cells that escape the treatment. It can also create radiation “hot spots” with risk of inducing local toxicity, although this is not regarded as a major problem with an α‐emitter due to its short range in tissues. Microparticle residue was observed at necropsy in the biodistribution studies here and can indicate a suboptimal dispersion. Adhesions caused by particle residue were never observed in our studies, in contrast to what was seen when biodegradable polymer microparticles (poly[lactic‐co‐glycolic acid]) were administered IP in mice.^52^ On the other hand, therapeutic radiopharmaceuticals based on small molecules like monoclonal antibodies often show heterogeneous distribution in solid tumors,^2^ which can also directly impact the therapeutic efficacy.

Taking possible heterogeneous microparticle distribution in the peritoneal cavity into account, a limited release of ^224^Ra and daughters from the microparticles might be an advantage. A controlled release of radionuclides from the microparticles can possibly contribute to a more uniformly absorbed dose, given that it does not lead to any unwanted toxicities as discussed previously. This aspect is especially interesting because it was found that the ^224^Ra release correlated with the amount of carrier administered. Another aspect is the fate of the gaseous ^220^Rn daughter, as it may diffuse away from the microparticles after decay of ^224^Ra. Because of the short half‐life of ^220^Rn (56 s) it will probably not redistribute from the cavity to a large extent before decay but might also contribute to reduce dose inhomogeneity. It is still unresolved whether ^220^Rn diffusion and ^224^Ra release from the microparticles actually influence the therapeutic efficacy of the ^224^Ra‐labeled CaCO_3_ microparticles. Further studies are needed to confirm or deny this hypothesis, but given the adjustable stability of the product, it should be possible to find a carrier dose that gives optimal therapeutic efficacy and thus shed light on whether some ^224^Ra release is beneficial to create a dose smoothening effect by counteracting heterogeneity in the microparticle distribution.

Another important aspect to address is the radiation safety related to possible release of ^220^Rn during preparation of the ^224^Ra‐labeled CaCO_3_ microparticles. As with all procedures involving open sources of α‐emitting radionuclides, precautions must be followed to avoid inhalation or ingestion. All handling of open sources should therefore be performed in either a biosafety bench or in a glove‐box under negative pressure to protect the worker. A study on the safety aspects of using ^224^Ra for medical applications is currently ongoing. The preliminary results indicate that there is no significant leakage of ^220^Rn when the ^224^Ra‐labeled CaCO_3_‐microparticle solutions are contained in closed vials and the diffusion into air from open vials was much lower than from solutions of free ^224^Ra.^53^

When proposing use of an internal α‐emitter for cancer treatment, it is important to choose a radionuclide with physical properties apt for the application. Intraperitoneal therapy with α‐emitters has shown significant potential in murine models previously, mainly with short‐lived nuclides coupled to monoclonal antibodies.^54^, ^55^, ^56^, ^57^, ^58^ With longer lived nuclides, an unfavorable biodistribution in mice without tumors was found after IP injection of ^227^Th coupled to trastuzumab.^59^ Because of the small size of the antibodies, the radioactivity cleared relatively rapidly from the peritoneal cavity. Studies have also been performed with short‐lived ^211^At bound to polystyrene microparticles. The obtained results were promising, with favorable biodistribution^60^ and cure rates.^61^ By using microparticles as carriers for radionuclides, a longer‐lived radionuclide, like, eg, ^224^Ra, might be advantageous. Because of the potential of increased IP residence time with microparticles compared with antibodies or other small molecules, it can be possible to deliver a higher radiation dose IP than with a short‐lived nuclide. It will therefore be interesting to evaluate the therapeutic effect of the ^224^Ra‐labeled microparticles described here in future studies and see if there is a therapeutic gain with our carrier labeled with a radionuclide with longer half‐life than ^211^At, which, in addition, exhibits some release of radionuclides from the particles that can counteract possible dose heterogeneity.

Another difference between our proposed ^224^Ra‐CaCO_3_ microparticles and the ^211^At‐microparticles is the composition of the carriers. Polystyrene is a nonbiodegradable material, whereas CaCO_3_ is composed of 2 components readily available in the body and will slowly decompose to Ca^2+^ and CO_3_^2−^. Because of the soluble nature of the microparticles, ^223^Ra and ^225^Ra were considered to have too long half‐lives to be a proper fit. By utilizing ^223^Ra or ^225^Ra, more radium would be released systemically because of their longer half‐lives, which is a potential risk for toxicities in normal tissues.

Altogether, the results from the current study, reviewed in conjunction with existing literature, indicate that there is a good fit between the physical properties of ^224^Ra, the CaCO_3_ microparticle carrier, and the intended treatment mode. Future work is warranted to determine the optimal size and dose of the microparticles. Exploring alternative formulations for injection to obtain a less heterogenous microparticle distribution in the peritoneal cavity is also of interest. In mice studies, 150 to 1000 kBq/kg of ^224^Ra‐labeled CaCO_3_ microparticles seems to be therapeutically relevant and well‐tolerated.^62^ If this, together with the microparticle doses of 5 to 25 mg used in this study, is to be translated into human equivalent doses according to the procedure recommended by the FDA,^63^ doses of 0.5 to 3 MBq/m^2^ and 0.6 to 3 g/m^2^ are obtained. By using an average body surface area of 1.79 m^2^ found in a study of adult cancer patients,^64^ this corresponds to 1 to 5.4 MBq ^224^Ra adsorbed onto 1 to 5.4‐ g CaCO_3_ microparticles.

## 5 CONCLUSIONS

Calcium carbonate microparticles labeled with ^224^Ra on their surfaces were prepared efficiently and with high yields. The retention of ^224^Ra and daughter ^212^Pb by the microparticles in vitro was also high, indicating that the radiolabeled particles may have a shelf‐life which allows sufficient time for centralized production, quality control, and shipment to the end user. The in vivo biodistribution studies suggest that the ^224^Ra‐labeled CaCO_3_ microparticles remain in the peritoneal cavity with modest distribution of ^224^Ra systemically, when administered at a relevant microparticle dose. An especially interesting observation was that release of ^224^Ra from the microparticles in vivo correlated with the amount of administered particles. In conclusion, the α‐emitting microparticles have properties that could make them a promising new modality for intracavitary cancer therapy.

## DISCLOSURES

SW, ISJ, EN, TBB, and ØSB are employed and own stock in Oncoinvent AS. MM was employed by Oncoinvent AS at the time when her contribution to the research article occurred. RHL is chairman of the board of Oncoinvent AS and a shareholder. Oncoinvent AS holds intellectual property rights to the presented technology (patent name: radiotherapeutic particles and suspensions. Patent number: US9539346 B1).

## Supporting information

Figure S1 Biodistribution of the daughter nuclide ^212^Pb in athymic nude mice, 1, 4, and 7 days following intraperitoneal injection of ^224^Ra‐labeled first generation CaCO_3_ microparticles (A) and dissolved ^224^RaCl_2_ (B). The data are shown as a bar representing the median ^212^Pb activity per mass unit in addition to the individual data points for each mouse. The injected activity has been normalized to 10 kBq per mouse.

Supporting info item
