# Supplementary material for: Ra‐224 labeling of calcium carbonate microparticles for internal α‐therapy: Preparation, stability, and biodistribution in mice
Source: J Labelled Comp Radiopharm. 2018 Mar 12;61(6):472–86. doi: 10.1002/jlcr.3610 (PMC6001669; doi:10.1002/jlcr.3610)
Supplement: Supplementary file 1 — Figure S1 Biodistribution of the daughter nuclide 212Pb in athymic nude mice, 1, 4, and 7 days following intraperitoneal injection of 224Ra‐labeled first generation CaCO3 microparticles (A) and dissolved 224RaCl2 (B). The data are shown as a bar representing the median 212Pb activity per mass unit in addition to the individual data points for each mouse. The injected activity has been normalized to 10 kBq per mouse. [file JLCR-61-472-s001.docx]

Figure S1 Biodistribution of the daughter nuclide ^212^Pb in athymic nude mice, 1,4 and 7 days following intraperitoneal injection of ^224^Ra-labeled first generation CaCO_3_ microparticles (A) and dissolved ^224^RaCl_2_ (B). The data is shown as a bar representing the median ^212^Pb activity per mass unit in addition to the individual data points for each mouse. The injected activity has been normalized to 10 kBq per mouse.
