# Supplementary figures and images for: Ra‐224 labeling of calcium carbonate microparticles for internal α‐therapy: Preparation, stability, and biodistribution in mice
Source: J Labelled Comp Radiopharm. 2018 Mar 12;61(6):472–86. doi: 10.1002/jlcr.3610 (PMC6001669; doi:10.1002/jlcr.3610)

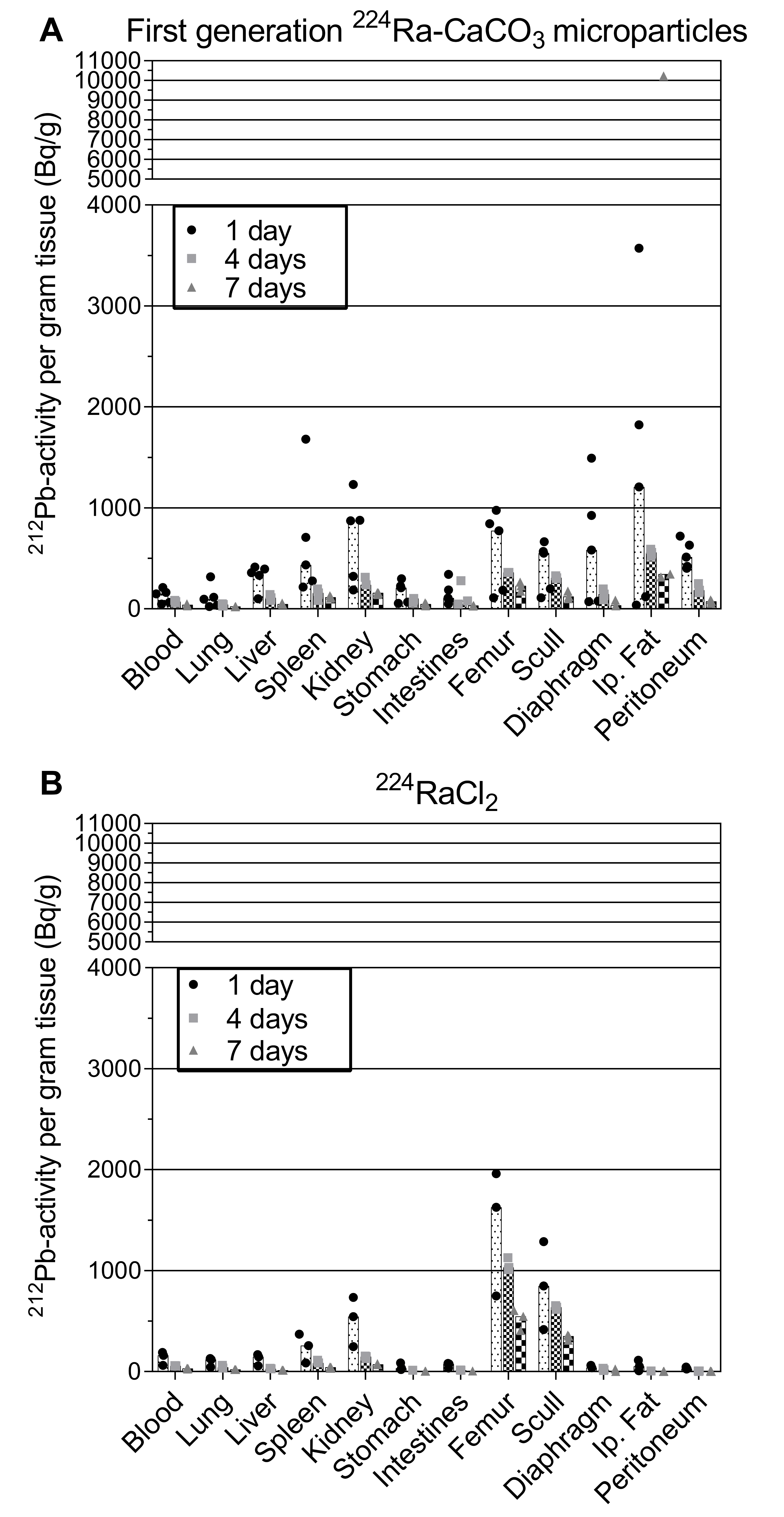

Supplement: Supplementary file 2 — Supporting info item [file JLCR-61-472-s002.tif]
